# Solid Lipid Nanoparticles Embedded Hydrogels as a Promising Carrier for Retarding Irritation of Leflunomide

**DOI:** 10.3390/gels9070576

**Published:** 2023-07-14

**Authors:** Hawra Mohammed Alhelal, Sidharth Mehta, Varsha Kadian, Vandita Kakkar, Himanshi Tanwar, Rekha Rao, Bandar Aldhubiab, Nagaraja Sreeharsha, Pottathil Shinu, Anroop B. Nair

**Affiliations:** 1 Department of Pharmaceutical Sciences, College of Clinical Pharmacy, King Faisal University, Al-Ahsa 31982, Saudi Arabia; baldhubiab@kfu.edu.sa (B.A.); sharsha@kfu.edu.sa (N.S.); anair@kfu.edu.sa (A.B.N.); 2 Department of Pharmaceutical Sciences, Guru Jambheshwar University of Science and Technology, Hisar 125001, India; sidharthmehta479@gmail.com (S.M.); kadyanvarsha313@gmail.com (V.K.); tanwarhimanshi@gmail.com (H.T.); 3 Department of Pharmaceutics, University Institute of Pharmaceutical Sciences, Panjab University, Chandigarh 160014, India; itakakkar@yahoo.co.in; 4 Department of Pharmaceutics, Vidya Siri College of Pharmacy, Off Sarjapura Road, Bangalore 560035, India; 5 Department of Biomedical Sciences, College of Clinical Pharmacy, King Faisal University, Al-Ahsa 31982, Saudi Arabia; spottathail@kfu.edu.sa

**Keywords:** Leflunomide, hydrogels, inflammation, lipid carrier system, topical

## Abstract

Leflunomide (LEF), a disease-modifying anti-rheumatic drug, has been widely explored for its anti-inflammatory potential in skin disorders such as psoriasis and melanoma. However, its poor stability and skin irritation pose challenges for topical delivery. To surmount these issues, LEF-loaded solid lipid nanoparticles (SLNs) integrated with hydrogels have been developed in the present investigation. SLNs developed by microemulsion techniques were found ellipsoidal with 273.1 nm particle size and −0.15 mV zeta potential. Entrapment and total drug content of LEF-SLNs were obtained as 65.25 ± 0.95% and 93.12 ± 1.72%, respectively. FTIR and XRD validated the successful fabrication of LEF-SLNs. The higher stability of LEF-SLNs (*p* < 0.001) compared to pure drug solution was observed in photostability studies. Additionally, in vitro anti-inflammatory activity of LEF-SLNs showed good potential in comparison to pure drugs. Further, prepared LEF-SLNs loaded hydrogel showed ideal rheology, texture, occlusion, and spreadability for topical drug delivery. In vitro release from LEF-SLN hydrogel was found to follow the Korsmeyer-Peppas model. To assess the skin safety of fabricated lipidic formulation, irritation potential was performed employing the HET-CAM technique. In conclusion, the findings of this investigation demonstrated that LEF-SLN hydrogel is capable of enhancing the photostability of the entrapped drug while reducing its skin irritation with improved topical delivery characteristics.

## 1. Introduction

Inflammation occurs as a response of the immune system to various hazardous stimuli, like infections, damaged toxic cell compounds and radiation [1]. Thus, it represents a vital in-built defense mechanism of the body [2]. Further, in acute inflammatory responses, molecular and cellular episodes help in impending injuries efficiently. These episodes further contribute to the restoration of tissue homeostasis and hence, resolve acute inflammations. Additionally, if acute inflammations become uncontrolled, it may result in various chronic diseases [3]. Usually, synthetic anti-inflammatory drugs have been employed for inhibiting or suppressing inflammations [4]. Leflunomide (LEF) is one such synthetic drug that possesses immunomodulatory and anti-inflammatory potential. It is regularly employed for the management of chronic inflammatory and autoimmune disorders such as psoriasis, skin cancer and rheumatoid arthritis. However, its administration suffers from several limitations such as poor solubility, low permeability, light sensitivity, and irritation [5]. Withal, its oral administration is associated with gastrointestinal problems such as nausea, dyspepsia, abdominal pain, and oral ulceration [6]. Therefore, a topical administration strategy may assist in bypassing delivery route associated problems of LEF. Further, topical application of drugs could also allow their higher concentrations at the inflammation sites. Apart from minimizing systemic exposure, this strategy may help in reducing drug-related adverse effects [7]. Another focus of the researchers is to improve the above-mentioned physicochemical issues of LEF. Keeping this in view, over the past decades, various novel strategies such as niosomes [5], nanoparticles [8], nanoemulgels [9], nanostructured lipid carriers [10,11], and solid lipid nanoparticles (SLNs) [12] have been prominently explored.

Lipid nanocarriers have gained popularity for topical administration owing to their good biocompatibility, lesser toxicity, and closeness to skin composition [13,14,15]. Lipids like triglycerides, partial glycerides, steroids, fatty acids, and waxes have been commonly used for the preparation of various lipid-based nanocarriers [16,17]. This carrier system offers improved skin permeation as well as retention of the drug, reduced dose, skin targeting, controlled release, enhanced pharmacological potential, and hence, better patient compliance [13,14]. Among lipid nanocarriers, SLNs represent a promising approach for the delivery of lipophilic drugs like LEF [18]. SLNs possess rigid core composed of hydrophobic lipids, which are surrounded by a phospholipid monolayer. These lipidic nanoparticles maintain their shape both at body temperature and at room temperature [19]. Aside from the above mentioned merits, SLNs can be utilized for skin targeting by augmenting the drug penetration evenly at the targeted site. Additionally, these nanocarriers help the entrapped moiety to attain stability and extend its release [20]. Due to their non-irritating and non-toxic qualities, SLNs are also suitable for application on inflamed skin [21].

Alternately, the small size of SLNs ensures their intimate contact with the stratum corneum enhancing its occlusive as well as bioadhesive characteristics, which play a vital role in topical delivery. The topical application of SLNs is efficient in making a thin film over the skin layer owing to their occlusive features [22]. SLN dispersions can also be integrated with commonly used dermal vehicles like creams and hydrogels. Among these, hydrogels are generally preferred because of their elegance, non-sticky nature, moisturizing capability, controlled release characteristics, improved skin targeting, flexibility in tailoring swelling behavior, and good tissue compatibility [23,24]. Additionally, spreadability, good wettability, viscoelasticity, softness, and good skin adherence make them superior carrier systems [25].

Based on the effectiveness of SLNs in inflammatory disorders, the present research was carried out to fabricate LEF-SLN loaded hydrogel for topical delivery. Herein, the aim of our study was to reduce skin irritation and improve the photostability of LEF-SLNs. This is a first of its kind study, which assessed the improvement in photostability and decreased irritation potential of LEF by encapsulating it in SLN-based hydrogel. Formulations were suitably characterized and evaluated for their release behavior, photostability, and anti-inflammatory potential. Subsequently, LEF-loaded SLNs were amalgamated with Carbopol hydrogels to augment their characteristics for topical application. Subsequently, formulated hydrogels were checked for pH, occlusive analysis, viscosity, spreadability, and texture. In addition, in vitro irritation potential of these hydrogels was also assessed.

## 2. Results and Discussion

SLNs are known to combine the merits of liposomes, emulsions, and polymeric nanoparticles [26,27]. The composition of blended key pharmaceutical ingredients can protect the entrapped moieties against environmental factors (sunlight), and chemical oxidation while regulating the drug release profile [17,27,28,29]. This nanosystem can easily be embedded in hydrophilic carrier systems like hydrogels. In the current study, SLN integrated hydrogel has been proposed for the topical application of LEF. The drug, LEF has poor water solubility (less than 40 μg/mL) with a log *p* value of 2.8 [30]. These physical characteristics and structure of this active moiety make it ideal for encapsulation in SLNs. Drug delivery from SLNs integrated into hydrogel seems to be advantageous as compared to delivery from classical dermatological and topical formulations [22]. Therefore, LEF-SLN-loaded hydrogels have been formulated to avoid the photodegradation of this active moiety, delay its release, and minimize associated side effects. Moreover, the irritation potential of LEF was also anticipated to be reduced by this novel carrier system.

LEF-loaded SLNs were formulated successfully by microemulsion method employing Tween 80, Compritol^®^ 888 ATO, and Phospholipon^®^ G90. It is worth mentioning here that microemulsions generally possess a high concentration of surfactants (30% or above), which may result in skin irritation if administered topically [31]. Integration with plain hydrogel reduces the surfactant concentration in the final formulation thereby helping in addressing the above mentioned challenges. Further, Compritol^®^ 888 ATO was selected as solid lipid in the fabricated SLNs as it helps in formulating stable dispersions with small globule size [32].

### 2.1. Characterizations of LEF-SLNs

#### 2.1.1. Particle Size Distribution, Zeta Potential, and PDI

**Figure 1:**
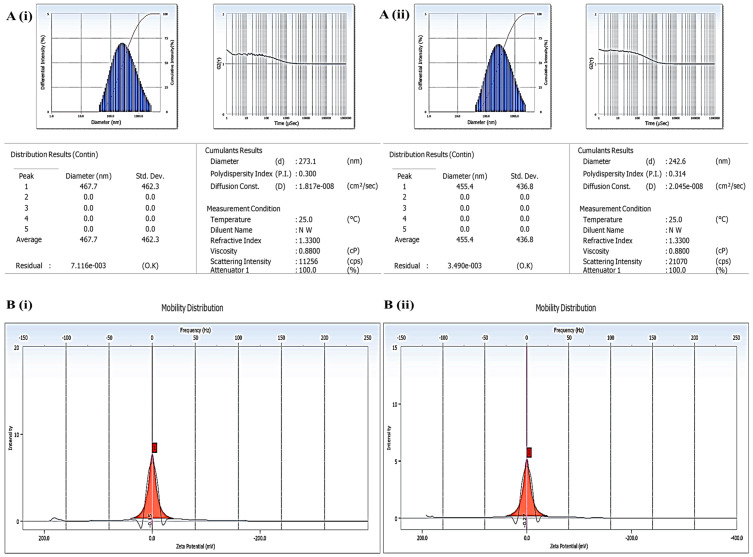
(**A**) Particle size and particle size distribution of (**i**) LEF-SLNs, (**ii**) blank SLNs and (**B**) Zeta potential of (**i**) LEF-SLNs, (**ii**) blank SLNs.

The particle size is a crucial parameter in SLNs that affects the loading, permeability, and release characteristics of loaded drugs [33]. Hence, it is accountable for the penetration of SLNs into deeper skin annexes [23]. For efficient topical delivery, the average particle size of fabricated SLNs should be <350 nm [34]. Herein, the mean size of LEF-SLNs was found 273.1 nm with a PDI of 0.300. Low PDI indicated a narrow particle size distribution of drug-loaded SLNs, while the average size of the blank formulation was 242.6 nm with a PDI of 0.314 (Figure 1). In addition, it was observed that entrapment of the Leflunomide did not remarkably alter the size of SLNs. Moreover, the selected surfactant as well as co-surfactant concentration probably reduced surface tension resulting in the production of smaller nanoparticles in both blank and LEF-SLNs.

Zwitter ionic (phospholipidic surfactant, Phospholipon^®^ 90 G) and non-ionic surfactant (Tween 80) combination resulted in the zeta potential of −0.15 mV and −0.27 mV, for LEF-SLNs and blank SLNs, respectively. This low zeta potential is responsible for imparting sufficient stabilization to fabricated SLNs. Tween 80 gets easily adsorbed and gives steric stabilization resulting in the low zeta potential of nanoparticles. Thus, SLNs were found stable owing to their low zeta potential values [23,35].

#### 2.1.2. Surface Morphology

**Figure 2:**
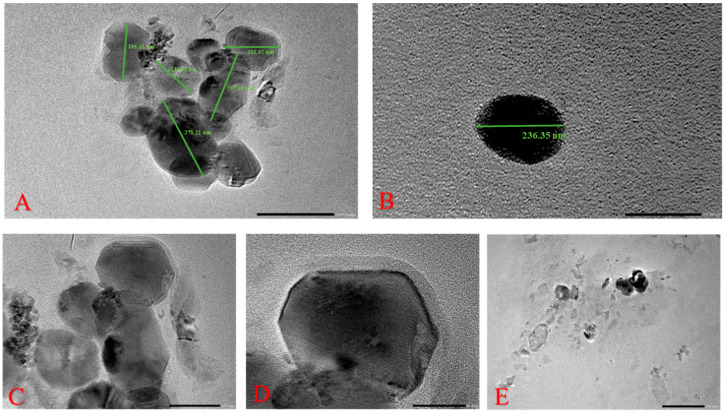
Transmission electron microscopy of LEF-SLNs (**A**) illustration of particle size distribution histogram along with the Gaussian profile (solid line) (**B**–**E**) illustration of LEF-SLN particles.

**Figure 3:**
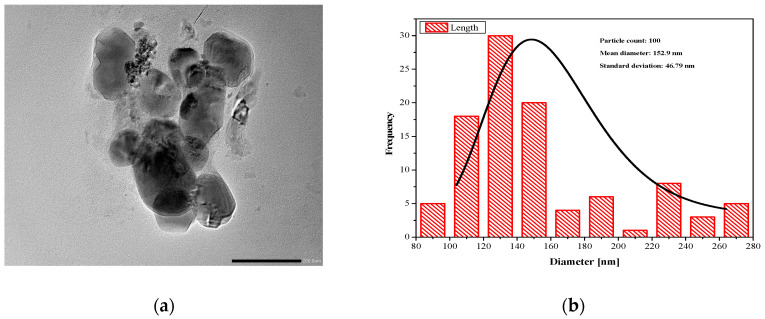
(**a**) Transmission electron microscopy image of LEF-SLNs referred for histogram (**b**) Particle size distribution (number distribution) obtained by TEM for LEF-SLNs. (The solid line represents Gaussian profiles from the mean diameter and ±SD, as computed from 100 particles).

To examine the internal structure and topography of LEF-SLNs, TEM analysis was carried out [23,36]. As can be seen in Figure 2, particles of nanoformulation are almost spherical with a smooth surface with particle size in the nano range (116–298 nm). LEF-SLNs particles were found segregated without any irregularity in the structure [32]. Accurate particle size determined using ImageJ software. Figure 3 represents the particle size distribution of more than 100 particles which was observed as 152.90 ± 46.79 (d/nm).

#### 2.1.3. Total Drug Content and Entrapment Efficiency

The total drug content of fabricated LEF-SLN dispersions was 93.12 ± 1.72%, indicating an insignificant loss during the preparation of formulation. The entrapment efficiency of LEF-SLNs was measured employing the dialysis bag method and observed as 65.25% ± 0.95 (n = 6). This might be ascribed to the higher solubility of LEF in the chosen solid lipid [32].

#### 2.1.4. FTIR Spectroscopy

**Figure 4:**
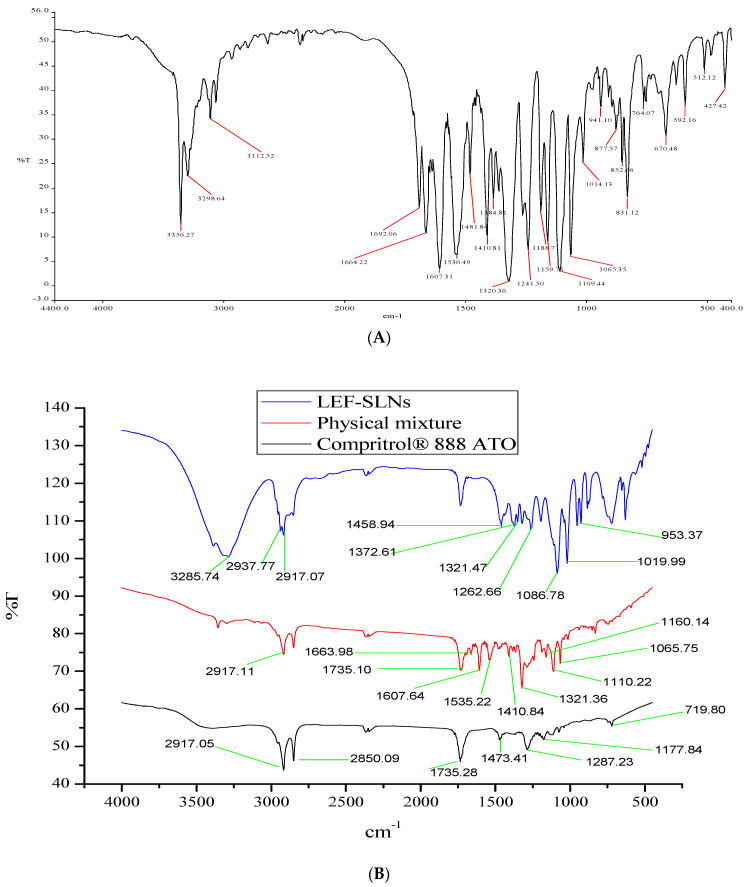
FTIR spectra of (**A**) LEF, (**B**) Overlay of Compritol^®^ 888 ATO, Physical mixture and LEF-SLNs.

FTIR spectra of pure LEF, Compritol^®^ 888 ATO, physical mixture and LEF-SLNs have been illustrated in Figure 4. Characteristic peaks of LEF appeared at 3356.27 cm^−1^, 3298.64 cm^−1^, 3112.52 cm^−1^, 1692.06 cm^−1^, 1607.31 cm^−1,^ and 1384 cm^−1^ which were attributed to NH– of amide, –C–H of the aromatic ring, –CH stretching vibration, HC=N–O of the isoxazole ring, C=O group of the amide fragment and –C–N stretching vibration, respectively [11]. Compritol^®^ 888 ATO characteristic FTIR peaks corresponding to C-H stretching and C-O stretching (Figure 4B) were analyzed at 2850.09 cm^−1^ and 1735.28 cm^−1^, respectively. Peaks between 719.60 and 1473.41 cm^−1^ were assigned to methylene groups [37]. FTIR spectrum of the physical mixture of LEF and Compritol^®^ 888ATO was represented in Figure 4B. No major change in characteristic peaks was observed indicating a lack of interaction between them and advocating their compatibility. Furthermore, the spectrum of LEF-SLN (Figure 4B showed the absence of characteristic peaks of LEF confirming its molecular dispersion in the formulation [38].

#### 2.1.5. XRD Analysis

**Figure 5:**
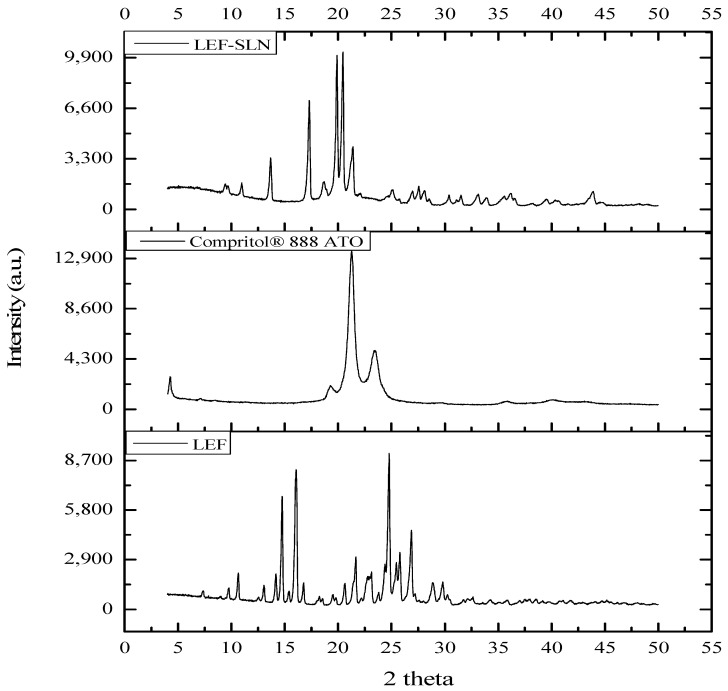
XRD pattern of LEF, Compritol^®^ 888 ATO and LEF-SLNs.

The XRD patterns of pure LEF, Compritol^®^ 888 ATO, and LEF-SLNs are illustrated in Figure 5. As per the diffraction analysis of pure drug, LEF is a highly crystalline compound with its characteristic peaks at 2θ 10.63, 14.75, 16.06, 21.65, 24.78, 25.80, and 26.87 degrees. Whereas, prominent peaks at 2θ 4.26, 21.26, 23.35, and 23.45 were noticed in Compritol^®^ 888 ATO due to lipid polymorphism [23,32]. The XRD pattern of LEF-SLNs represented characteristic peaks at 2θ 13.68, 17.30, 19.90, 2 0.46, and 21.38. The distinct peaks of pure LEF have not been observed in LEF-SLN, ascertaining the amorphous character of the developed carrier. This amorphous character was responsible for delayed release of LEF from LEF-SLNs [32,39]. There was 73.18%, 63.81%, and 37.02% crystallinity in Compritol^®^ 888 ATO, LEF, and LEF-SLN, respectively as determined by employing a curve-fitting process (OriginPro^®^ 2021). The crystallinity of pure LEF (63.81%) was found higher than LEF-SLNs (37.02%). The crystallite size of LEF (36.49 nm) was also observed bigger as compared to LEF-SLNs (23.59 nm).

### 2.2. Photostability Assessment

**Figure 6:**
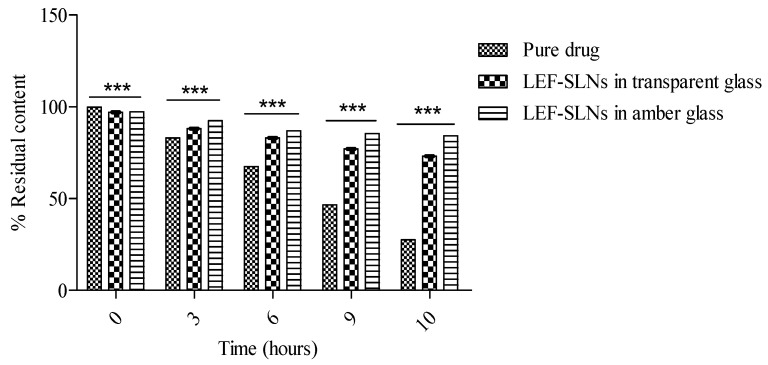
Photostability assessment of LEF in aqueous Tween 80 and SLN dispersions (in a transparent and amber color container). Data analysis was performed by Two way ANOVA, followed by Bonferroni post-tests. A statistically significant difference (*** *p* < 0.001) was observed.

The photostability study of drugs is generally carried out to determine any unacceptable changes due to light exposure [40]. This study was one of the key objectives of current research. The photostability study of LEF-loaded SLN dispersion was compared to pure LEF in aqueous Tween 80 through spectrophotometric measurements (by determining the percent degradation of the drug). The samples were analyzed before irradiation and after irradiation (diluting appropriately using ethanol) at increasing periods. Figure 6 shows graphs of the photodegradation of LEF-SLN dispersions and pure LEF (in amber colored as well as transparent containers), after irradiation time. It was observed that the stability of LEF-SLN dispersions (in amber colored as well as transparent glass) against sunlight was enhanced with respect to pure LEF samples. In the current study, above 80% of LEF content (84.15 ± 1.37% in the amber-colored container) was observed after irradiation for 10 h in SLNs whereas, only 27.56 ± 1.82% of residual LEF content was found in aqueous Tween 80. Non-significant changes were observed for pH and drug encapsulation of SLN dispersions in amber color and transparent glass (Table 1). This remarkable enhancement of LEF photostability advocated the protective potential of the SLN delivery system. In nutshell, it was concluded that SLN augmented the photostability of LEF and can be used as a promising strategy for protection of photolabile drugs.

### 2.3. In Vitro Anti-Inflammatory Assay

**Figure 7:**
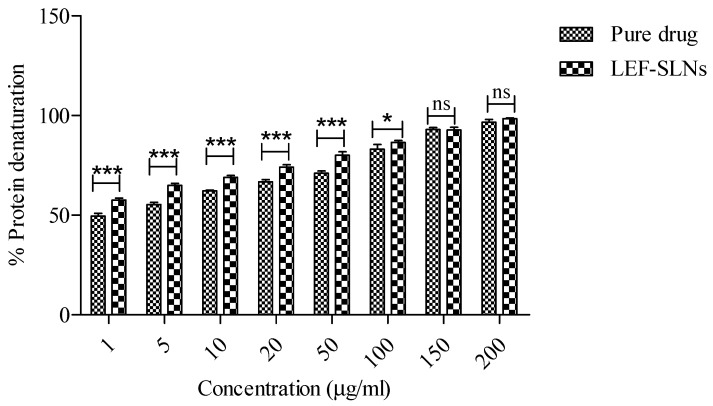
Percent protein denaturation of LEF and LEF-SLNs dispersion. All the values are presented in triplicate (mean ± SD); * represents *p* < 0.05, *** represents *p* < 0.0001, and ns represents non-significant analysis via Two way ANOVA, followed by Bonferroni post-tests.

The anti-inflammatory potential of LEF is a key contributing factor in resolving various inflammatory skin conditions like psoriasis, acne, eczema, atopic dermatitis, and skin cancer [41]. Hence, we have evaluated the anti-inflammatory potential of LEF and LEF-loaded SLNs employing in vitro bovine serum albumin denaturation assay. Both LEF and its formulation showed anti-inflammatory potential in a dose-dependent manner. Pure drug and LEF-SLN dispersions exhibited protein denaturation inhibition from 49.56 ± 1.39% to 96.67 ± 1.33% and 57.56 ± 1.02 to 98.44 ± 0.38%, respectively (sample concentrations: 1 to 200 µg/mL) (Figure 7). From the outcomes of this assay, it has been concluded that LEF-SLN dispersions exhibited greater anti-inflammatory potential as compared to LEF.

### 2.4. Characterization of LEF-SLN-Based Hydrogels

The developed SLN hydrogels were white in color having a good appearance and smooth texture without any phase separation. Further, the formulations were found translucent, consistent, and homogeneous with no coarse particles (Table 2). The pH value of the LEF-SLN hydrogel was observed as 6.66 ± 0.04, which is closer to the human skin pH, hence the minimal risk of skin irritation is expected [42].

**Figure 8:**
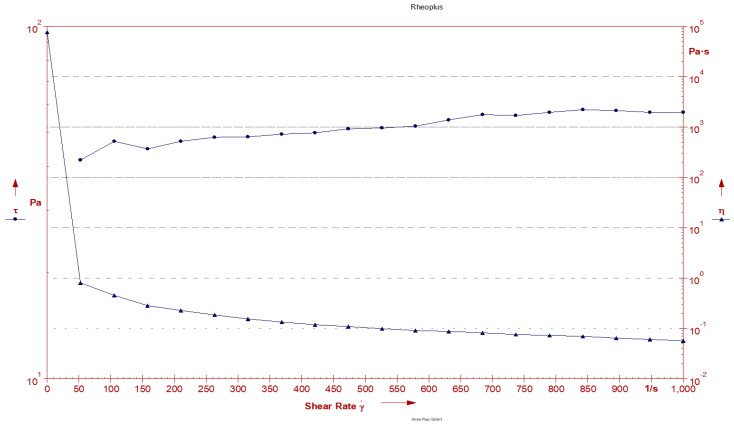
Plot of shear rate vs shear stress (τ) and shear rate vs viscosity (η) for LEF-SLN hydrogel.

It is well known that the rheology of topical formulations has a remarkable effect on their contact time, spreadability, and skin retention [43,44]. Therefore, the viscosity profile of LEF-SLN hydrogel (0.025% *w*/*w*) was studied. The apparent viscosity of LEF-SLN hydrogel was obtained as 97.5 mPa·s at a constant shear rate of 10 s^−1^ and temperature of 30 °C. Figure 8 illustrated that there is variation in viscosity and shear stress of the developed SLN hydrogel with increasing shear rate. The n values (representing the shear thickening or shear thinning of the hydrogel) were found <1 (n = 0.156), corroborating the shear thinning property of LEF-SLN hydrogel. The rheogram represented the viscosities at varying shear rates (Figure 8). The findings demonstrated a consistent decrease in viscosity with an increase in shear rate, suggesting that viscosity and shear rate are directly related. This pattern is desirable for topical formulations, as they are expected to remain thin during their application and otherwise thick [45,46].

**Figure 9:**
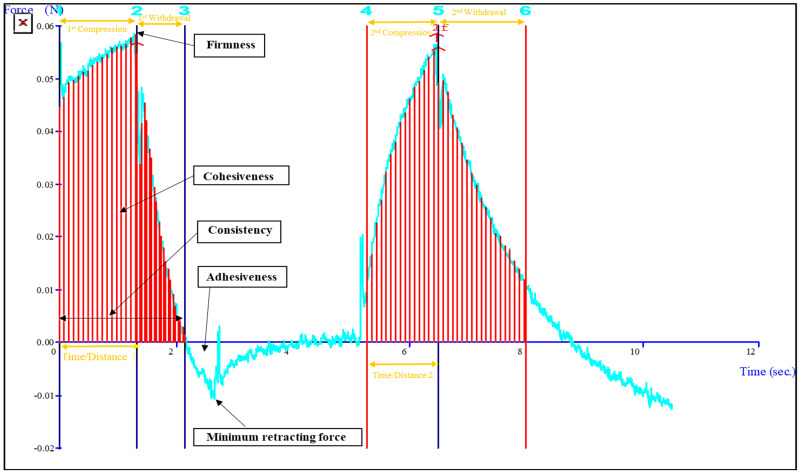
Graph representing texture analysis of LEF-SLN hydrogel.

Topical gel formulations need to possess favorable textural properties like hydrogel strength, consistency, stickiness, springiness, resilience, adhesiveness, and cohesiveness, as these factors directly affect the applicability, patient acceptability and therapeutic outcome [23]. The textural analysis of the LEF-SLN hydrogel was depicted in Figure 9. The results of consistency, firmness, springiness, index of viscosity, resilience, cohesiveness, and adhesiveness were found to be 0.898 N·s, 0.056 N, 89.13%, 0.057 N·s, 0.025 N·s, 1.017 N·s, and 0.02 N·s, respectively. The results showed that LEF-SLN hydrogel has sufficient strength, spreadability, bioadhesion, and extrusion.

### 2.5. Occlusion Testing

The findings of in vitro occlusive examination showed that the occlusion factor lies in the range of 10.20 to 85.13 for LEF-SLN hydrogel, blank SLN hydrogel, and LEF hydrogel formulation (Table 3). Significant differences were observed among the results obtained at chosen time intervals (24 and 48 h; Table 3). Therefore, the present findings demonstrated that the occlusive behavior presented by LEF-SLN hydrogel adds to its merits for topical application.

At the end of 48 h, the % water loss from filter paper for LEF hydrogel (54.67 ± 0.32) was 5.40 times more than LEF-SLN hydrogel (10.13 ± 0.81) (Table 3). Escalated occlusion factor for LEF-SLN hydrogel (85.13 ± 1.19) was ascribed to the emollient potential of the lipids present in this SLN integrated hydrogel as compared to the LEF hydrogel (19.79 ± 0.46) (Table 3). The slight occlusive behavior showed by LEF hydrogel is due to its composition, which is responsible for formation of the thin film. Howbeit, this occlusion value was remarkably low (*p* < 0.001) than blank hydrogel or LEF-loaded SLNs. It is interesting to highlight that the maximum occlusion factor was exhibited by SLN-loaded hydrogels, regardless of the integration of the drug. Thus, these findings would help in mitigating dryness, itching, and scaling commonly associated with inflammatory skin disorders.

### 2.6. Spreadability

**Figure 10:**
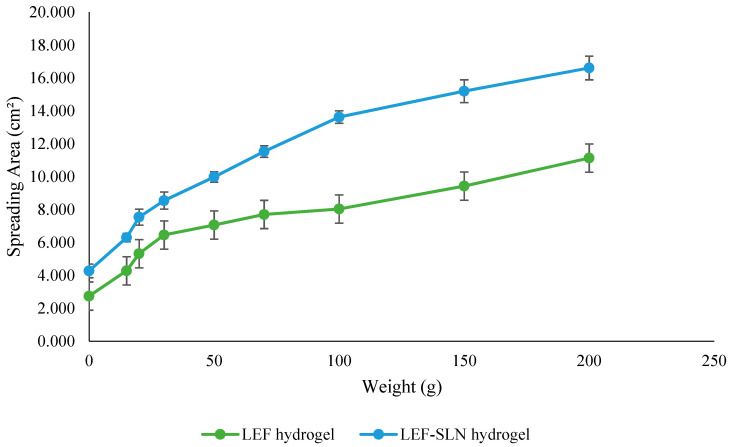
Spreadability study of LEF hydrogel and LEF-SLN hydrogel determined by parallel plate method.

The consistency of topical delivery products can be investigated through spreadability which plays a key role in ease of application as well as the accurate dosage delivery to the target site [47]. The spreadability behavior of LEF-SLN and LEF hydrogel is represented in Figure 10. The initial spreading area for LEF-SLN hydrogel and LEF hydrogel was found to be 4.28 ± 0.42 cm^2^ and 2.75 ± 0.44 cm^2^, respectively. On application of weight 200 g, the spreading area was found 16.62 ± 0.72 cm^2^ and 11.14 ± 0.34 cm^2^ for LEF loaded SLN hydrogel and LEF hydrogel, respectively. These results exhibited that LEF hydrogel was found to possess lesser spreadability with respect to LEF-SLN hydrogel (Figure 10). Integration of LEF-SLN dispersion with Carbopol hydrogel resulted in an increased spreadability making it more suitable for dermal application.

### 2.7. Drug Release

**Figure 11:**
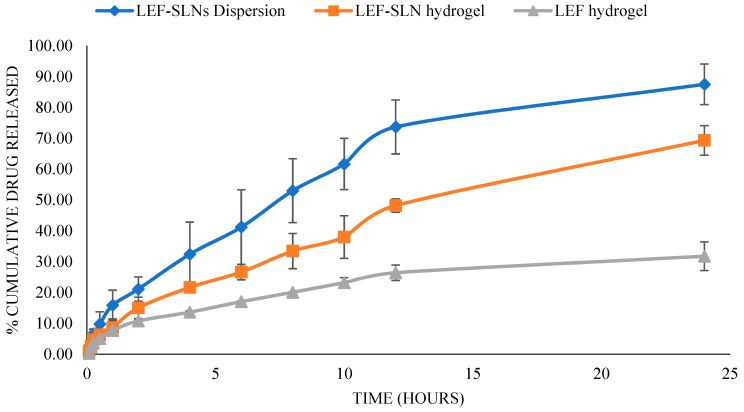
In-vitro release behavior of LEF-SLN dispersions, LEF hydrogel, and LEF-SLN hydrogel (Mean ± SD, n = 3).

The in vitro release behavior of fabricated LEF-SLN dispersion, LEF-SLN hydrogel, and LEF hydrogel formulation in phosphate buffer saline (pH 7.4) is presented in Figure 11. A rapid release pattern was observed for LEF-SLN dispersion, while the release was low and comparatively delayed for LEF-SLN hydrogel. Cumulative drug release from LEF-SLN hydrogel and LEF hydrogel was found 69.31 ± 4.79% and 31.77 ± 4.63%, respectively whereas, for LEF-SLN dispersions, it was 87.51 ± 6.59% in 24 h. In case of LEF-SLN dispersion, surfactant in its aqueous phase might have increased drug solubility and consequently enhanced its release. While LEF-SLN hydrogel presented a biphasic profile with burst release in the first 2 h, followed by delayed release over 24 h. The initial fast drug release observed here might be owing to free LEF present on the surface of nanoparticles. Further, therapeutically, the burst release pattern can be assumed to be beneficial for initial therapeutic action. Later, a delayed release of remaining LEF from LEF-SLN is advantageous in maintaining the therapeutic dose without the need for repeating the application [48]. Further, to assess the release pattern, the release data obtained (LEF-SLN dispersion, LEF hydrogel, and LEF-SLN hydrogel) was examined using various kinetic models such as zero order, first order, Korsmeyer-Peppas and Higuchi models. The higher correlation coefficient (r^2^ = 0.989) was obtained for LEF-SLN hydrogel by fitting the release data, suggesting Korsmeyer-Peppas as the best model. LEF-SLN hydrogel with n = 0.604 followed a non-fickian transport mechanism. Herein, the lipid cores of SLNs might have eroded slowly leading to release of the encapsulated LEF both via diffusion and/or dissolution. Further, for LEF-SLN dispersion first-order model (r^2^ = 0.982) was found best fit with an n value 0.560 ascertaining diffusion/dissolution mechanism. Higuchi model was observed best for LEF hydrogel with regression value 0.985 and n value 0.474, indicating diffusion release.

### 2.8. Irritation Behavior

**Figure 12:**
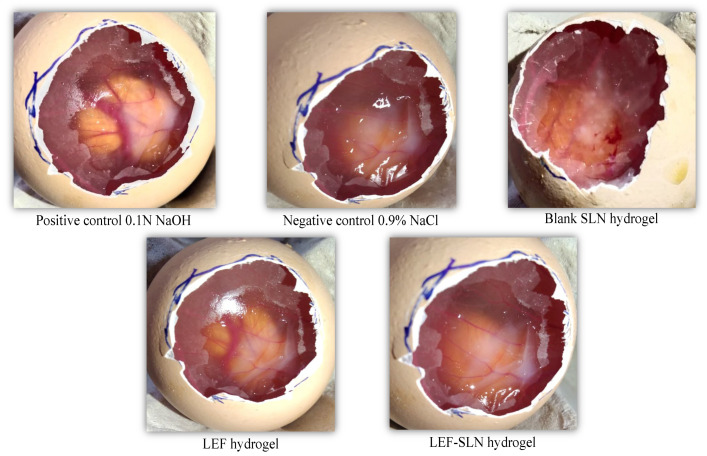
Irritation behavior of blank SLN hydrogel, LEF hydrogel, LEF-SLN hydrogel, negative and positive control using HET-CAM method (n = 3).

**Figure 13:**
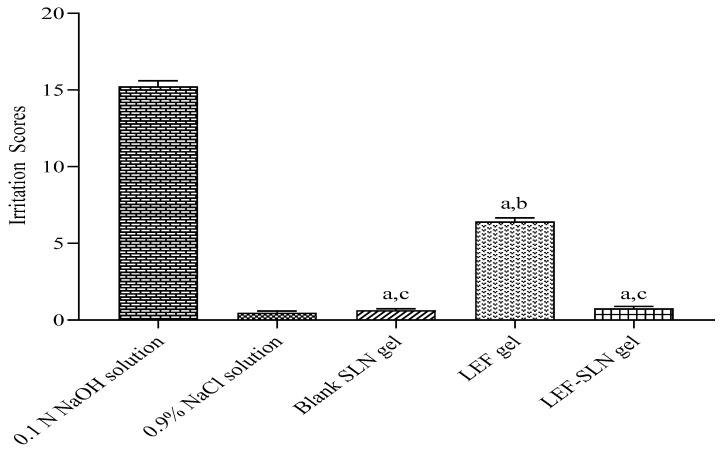
Irritation behavior of hydrogels (n = 3) performed via HET-CAM method, wherein ^a^ *p* < 0.001 (vs. 0.1 N NaOH solution), ^b^ *p* < 0.001 (vs. 0.9% NaCl solution), and ^c^ *p* < 0.001 (vs. LEF hydrogel) employing one-way ANOVA, followed by Tukey’s test (for multiple comparisons).

LEF-SLN hydrogel and LEF hydrogel were investigated for their irritation properties employing HET-CAM method. The CAM was exposed to each hydrogel sample and the irritation score was recorded (Figure 12 and Figure 13). The negative control (saline solution) showed no irritation (IP score: 0.46 ± 0.13), whereas, the positive control (0.1 N NaOH solution) instantly demonstrated severe irritation (IP score: 15.23 ± 0.37) with vascular hemorrhage, lysis, and blood coagulation. These outcomes were also found similar to our previous investigations [25,49]. These findings ascertained that HET-CAM method was appropriate for investigation of irritation behavior of samples (Figure 13). In another investigation by Savian et al., an IP score:17.43 ± 1.22 was observed for 0.1 N NaOH solution (positive control) [50]. On the other hand, the blank SLN hydrogel without LEF showed no irritation (IP score: 0.64 ± 0.09). An almost similar IP score (0.76 ± 0.14) was obtained for LEF-SLN hydrogel. Howbeit, LEF hydrogel was observed as a moderate irritant (IP score: 6.41 ± 0.25) based on the levels of hemorrhage and vasoconstriction. These results demonstrated that fabricated LEF-SLN hydrogel was efficient in minimizing the irritation of LEF moiety.

## 3. Conclusions

The present investigation emphasized the improvement of photostability and safety of LEF for topical application via fabrication of LEF-loaded SLNs and their post-integration into a hydrogel. Firstly, LEF-loaded SLNs have been successfully crafted by microemulsion technique employing solid lipid (Compritol^®^ 888 ATO), surfactant (Tween^®^ 80), and co-surfactant (Phospholipon^®^ G90). The lipidic formulation exhibited satisfactory physicochemical characteristics namely zeta potential, particle size, total drug content, and entrapment efficiency. Photo-degradation evaluation against sunlight showed higher stability of LEF-SLNs than LEF-aqueous dispersion. The in-vitro anti-inflammatory assay revealed an augmented anti-inflammatory effect with LEF-SLNs with respect to pure LEF-dispersion under similar conditions. After integration with hydrogel, LEF-SLNs exhibited acceptable outcomes in terms of appearance, pH, viscosity, texture, spreadability, and occlusivity. Release studies of the developed LEF-SLN hydrogel demonstrated delayed release of LEF bestowing its potential efficacy. The findings of irritation via the HET-CAM technique demonstrated that LEF-SLN-embedded hydrogel minimized drug irritation. Hence, depending on the above findings, SLN hydrogels are proposed as a potential vehicle for addressing photostability and irritation issues of LEF with improvement in the efficacy of the drug for topical application.

## 4. Materials and Methods

### 4.1. Chemicals

LEF and bovine serum albumin were obtained from Sigma-Aldrich (Milan, Italy). Compritol^®^ 888 ATO and Phospholipon^®^ 90 G were procured as a kind gift from Panacea Biotech (Lalru, India) and Phospholipid GmbH (Cologne, Germany), respectively. Carbopol^®^ 934 was provided by Central Drug House (P) Ltd. (New Delhi, India). Tween 80 was obtained from M/s Molychem Industries (Mumbai, India). Fertilized chicken (Cobb 500) eggs were acquired from Neelkanth Farms (Kurukshetra, India). Other reagents employed in this investigation were of pharmaceutical grade.

### 4.2. Development of LEF-SLNs

LEF-SLNs were fabricated employing the microemulsion technique with minor alterations [32]. In brief, Phospholipon^®^ 90 G (0.4% *w*/*w*), and Tween 80 (30% *v*/*v*) were added in water and were heated up to 82 °C to 85 °C (melting temperature of lipid). Separately, Compritol^®^ 888 ATO (300 mg) was also heated up to 82–85 °C. LEF (20 mg) was added to this lipidic phase, followed by the addition of a hot aqueous mixture (prepared above) along with an emulsifier (Compritol^®^ 888 ATO). Thus, obtained mixture was subsequently mixed under a magnetic stirrer to get a stable and clear yellow microemulsion. This hot microemulsion was further stirred (5000 rpm) with an equivalent amount of cold water (2 °C; 1.5 h). SLNs were formed in the aqueous phase due to crystallization of lipid particles present in the microemulsion. These LEF loaded SLNs were stored in glass vials in the refrigerator till further analysis.

### 4.3. Characterizations of LEF-SLNs

#### 4.3.1. Particle Size, Zeta Potential, and Polydispersity Index (PDI) Evaluation

Particle size and PDI were evaluated by photon correlation spectroscopy using Delsa^TM^ Nano C (Beckman Coulter, Brea, CA, USA) after appropriate dilution of SLN samples in Milli-Q water (1:50 *v*/*v*). Additionally, diluted samples were assessed for zeta potential based on electrophoretic mobility [51].

#### 4.3.2. Surface Morphology

Transmission electron microscopy (TEM) was employed for the morphological characterization of LEF-SLNs at Central Instrumentation Lab, Panjab University, Chandigarh, India. For TEM evaluation, the sample drop was adequately diluted (1:20) in double distilled water and was fixed on a copper grid (membrane coated). After drainage of excess fluid, the system was left to dry (at ambient temperature). Later, the samples were observed by HR-TEM (JEOL-JEM 2100 Plus, Tokyo, Japan) for assessing shape and size characteristics (irregularity or aggregation) [23,45]. The images were further assayed by ImageJ software (National Institute of Health, Bethesda, ML, USA).

#### 4.3.3. Total Drug Content and Entrapment Efficiency

The total drug content of fabricated LEF-SLN dispersions was evaluated by agitating the dispersion (1 mL) with ethanol: chloroform (1:1). This was centrifuged (at 5000 rpm) and filtered (0.45 µm membrane filter) to get a clear solution. Total drug content was computed using a standard curve plotted earlier employing a UV spectrophotometer at λ_max_ 267 nm.

The entrapment efficiency of fabricated LEF-SLN dispersions was analyzed by dialysis membrane (with aperture size 2.4 nm; MW cut off 12–14 kDa). The membrane was previously soaked in MilliQ water before use. SLN dispersion (1 mL) was taken in a dialysis bag, which was sealed from both ends and dialyzed into ethanol (50 mL) at 25 °C for 1 h. The drug amount determined in release media was analyzed spectrophotometrically. SLNs that remained in the dialysis tube were agitated using methanol: chloroform (1:1) mixture to compute LEF entrapped inside SLNs [52]. Entrapment efficiency was quantified by the following equation.

$$Entrapment\;efficiency\;\left(\%\right)=\frac{Entrapped\;drug\;content\;}{Actual\;drug\;content}\times 100$$

$$Drug\;loading\;\left(\%\right)=(\frac{Amount\;of\;drug\;added-Amount\;of\;free\;drug}{Amount\;of\;lipid\;added})\times 100$$

#### 4.3.4. FTIR Spectroscopy

FTIR spectra of pure LEF, Compritol^®^ 888 ATO, physical mixture (Compritol^®^ 888 ATO+LEF), and lyophilized LEF-SLNs were obtained from FTIR spectroscopy (60 MHz Varian EM 360, Perkin Elmer, Bridgeport Avenue, Shelton, CT, USA). The potassium bromide (KBr) pellet technique was employed for this analysis, in which the sample was blended with KBr and compressed into a pellet, and then employed for FTIR evaluation [53].

#### 4.3.5. X-ray Diffraction (XRD)

The pure LEF, Compritol^®^ 888 ATO, and lyophilized LEF-SLNs were performed for phase evaluation using XRD. This was carried out on an XPERT-PRO diffractometer system at 2θ angles from 4° to 50°. The copper was employed as anode material, and K_α_ and K_β_ radiations were observed at 45 kV (tube voltage) and 40 mA (tube current) at 25 °C [54].

The *crystallinity index* (*CI*) is generally determined and applied to explain the quantity of crystalline material in a substance. For comparison of *CI* from XRD peaks, we used the XRD deconvolution process. This process needs software (OriginPro^®^ 2021, Version 9.8) to differentiate crystalline and amorphous effects in the diffraction spectrum by the curve-fitting method [55]. *CI* is computed employing a given expression:

$$Crstallinity\;Index\;\left(CI\right)=\frac{Area\;of\;crystalline\;peaks}{Area\;of\;all\;peaks}\times 100$$

The crystallite size of LEF and LEF-SLNs was quantified from XRD spectra using the Scherrer formula:

$$t=\frac{\lambda \times 0.9}{\left(\beta \times cos\;\theta \right)}$$

where *t* represents crystallite size, *λ* stands for X-ray wavelength of radiation for Cu Kα (0.1542 nm), *β* denotes full-width at half maximum in radian, and *θ* is the angle of diffraction.

### 4.4. Photostability Analysis

The degradation of the drug in LEF encapsulated in SLNs and LEF in aqueous Tween 80 was evaluated by exposing these to sunlight. In brief, LEF (10.62 mg) in aqueous Tween 80 (20 mL) and LEF-SLN dispersions (20 mL) were laid bare for various time intervals (3, 6, 9, and 10 h) in sunlight in transparent, amber coloured vials. The drug content and pH were examined with fresh samples (t = 0) and at selected intervals. Drug content was assayed spectrophotometrically. Similarly, entrapment efficiency, pH, and total drug content of all samples at similar irradiation intervals were evaluated. LEF-loaded SLNs were spectrophotometrically analyzed after appropriate dilutions.

### 4.5. In-Vitro Anti-Inflammatory Assay

The protein denaturation study was performed by following the assay reported by Gunathilake et al., with minor changes [56]. The reaction mixture; at different concentrations had 1% *w*/*v* bovine serum albumin, (0.20 mL) and LEF or LEF-SLNs (2.00 mL) and 7.4 pH phosphate buffer saline (2.80 mL). After appropriate mixing, the samples were incubated (by placing them in a water bath heated for 15 min at 37 °C) and then, for 10 min at 70 °C. This mixture was cool down and the change in turbidity was analyzed at λ_max_ 660 nm employing GENESYS^™^ 180 UV-Vis Spectrophotometer [57]. The protein denaturation inhibition percentage was calculated employing the following equation:

$$\%\;inhibition\;of\;protein\;denaturation=100\times [1-\left(\frac{Absorbance\;of\;test\;sample}{Absorbance\;of\;control\;sample}\right)]$$

### 4.6. Incorporation of LEF-SLN into Hydrogel Base

For enhancing patient compliance, fabricated LEF-SLNs were integrated into the hydrogel delivery system. This was expected to enhance the spreadability of LEF-SLN dispersion when applied topically. Carbopol^®^ 934 hydrogel (1% *w*/*v*) was prepared in distilled water and allowed to swell overnight. Then, added triethanolamine dropwise to the obtained mixture with continuous stirring using a mechanical stirrer until a translucent hydrogel was obtained. Subsequently, LEF-SLNs dispersion (25 mL) was mixed slowly with obtained hydrogel in order to obtain 0.025% *w*/*w* LEF concentration in the fabricated hydrogel. LEF hydrogel was also formulated by integrating a similar 0.025% *w*/*w* of LEF concentration in Carbopol^®^ 934 (1% *w*/*v*) hydrogel, as in LEF-SLN hydrogel [32].

### 4.7. Visual Examination and pH Determination

LEF-SLNs dispersion, blank SLNs dispersion, blank SLN hydrogel, LEF hydrogel, and LEF-SLN loaded hydrogel were inspected visually for homogeneity, color, consistency, and the existence of any lumps. The pH of the obtained hydrogels was evaluated (in triplicate) by a digital pH meter (Controller Based pH system 362, Ahmedabad, India) at room temperature. Briefly, approximately 1 g of hydrogel was mixed in 10 mL of MilliQ water and the glass electrode was completely dipped into the hydrogel to determine pH [58].

### 4.8. Rheology and Textural Profile of Hydrogel

The viscosity of LEF-SLNs hydrogel was examined by rheometer (Rheolab QC, Anton Paar GmbH, Vienna, Austria). The hydrogel was kept in a sample holder, followed by the application of a shear rate (0.1–100 s^−1^). Recorded the viscosity and shear stress of the sample employing Rheoplus/32 version 3.40 software of the instrument at a fixed temperature (30 °C) [23].

LEF-SLN hydrogel was evaluated employing TA-XT2i^™^ Texture Analyzer (M/S Stable Micro Systems Ltd., Surrey, UK) to assess its stickiness and firmness. A standard beaker was loaded with about 50 mL of hydrogel formulation, avoiding the entry of air into the sample and ensuring the formation of a smooth top surface. A cylindrical ebonite probe (10 mm diameter) was a penetrated test in compression mode in this sample. A load cell weighing 5 kg a trigger force of 0.06 N, and a piercing depth of 4 mm were utilized in each test, with a velocity of 3 mm/second and pre-and post-test speed of 1 mm/second. The experiment was carried out at 25 °C, using Texture Expert (version 1.22, Stable Micro System, Haslemere, Surrey, UK). The LEF-SLN hydrogel was observed for consistency, firmness, index of viscosity, springiness, resilience, adhesiveness, and cohesiveness [59].

### 4.9. Occlusion Testing

In vitro occlusion test was carried out based on De Vringer’s technique [60]. The occlusive characteristics of blank SLN hydrogel, LEF-SLN hydrogel, and LEF hydrogel (n = 3) were examined using this study. The device was set up by using a 100 mL beaker having half the amount of distilled water and was wrapped with Whatman’s filter paper 41. Hydrogels were applied evenly on the filter paper surface with the help of a spatula and the whole system was kept at 32 ± 0.5 °C (imitating skin surface temperature) for up to 48 h. The beakers were weighed at the same time intervals (24 and 48 h) [61]. The percentage water loss was estimated and the occlusion factor (*F*) was computed using the given expression:

$$F=\frac{A-B}{A}\times 100$$

where *F* is denoted as the occlusion factor, *A* denoted as water loss without a sample (reference) and *B* stands for water loss from the sample.

### 4.10. Spreadability

The spreadability of hydrogels was analyzed by parallel plate process as mentioned previously [62]. In brief, 0.5 g LEF hydrogel or LEF-SLN hydrogel was kept on a glass slide and a second slide was placed concentrically on top. The diameter of the circle formed by hydrogel samples was observed initially. Selected weights (15, 20, 30, 50, 70, 100, 150, and 200 g) were placed gently on a top glass slide for 1 min and the hydrogel was allowed to spread. Then the spreadability of hydrogels was measured as the variation in the diameter of weights applied [59]. Outcomes were represented with regard to the spreading area using the following equation:

$$S=\pi d^{2}/4$$

where *S* is the spreading area (cm^2^) and *d* denotes the average diameter (cm) for every hydrogel tested.

### 4.11. In Vitro Drug Release

The amount of Leflunomide liberated was assayed employing a dialysis membrane with a 2.4 nm pore size (12–14 kDa cut-off molecular weight). The membrane was soaked in release media (pH 7.4, phosphate buffer) before the release study. LEF-SLN dispersions (2 mL), LEF-SLN hydrogel (4 g), or LEF hydrogel (4 g), each having 1000 µg of LEF were taken in a dialysis bag. The release medium, (phosphate buffer, 50 mL) was stirred continuously at 100 rpm with a magnetic stirrer (at 37 ± 0.5 °C temperature) throughout the experiment. Aliquots (5 mL) were taken at various periods and exchanged with an equal amount of new receptor fluid. The study was done for 24 h and then analyses were performed in triplicate at 267 nm using UV-spectrophotometry [32]. The drug release findings were further evaluated using various mathematical models to check the release kinetics [25].

### 4.12. Irritation Behavior

The irritation behavior of LEF-SLN hydrogel, blank SLN hydrogel, and LEF hydrogel, was examined via the hen’s egg test chorioallantoic membrane (HET-CAM) technique. Briefly, 10 days of fertilized chicken (Cobb 500) eggs (37.3 °C and 65% relative humidity) were selected for the study. On the 10th day, the blunt part of the eggs was elucidated by using the candling lamp. The eggs having a live embryo and air sac were marked for further testing [63]. Subsequently, the outer shell and white membrane were detached and the hydrogel sample was added to the CAM (n = 3/formulation). After 20 s, the hydrogel was washed with saline solution and the CAM was observed for 300 s. In this duration, the time required for coagulation, vasoconstriction, and hemorrhage was determined. Positive (0.1 N NaOH) and negative control (0.9% *w*/*v* NaCl solution) was also assessed [50].

The irritation potential (*IP*) score was calculated by using the equation:

$$IP=\frac{5\times (301-h)}{300}+\frac{7\times (301-v)}{300}+\frac{9\times (301-c)}{300}$$

where *h* represents hemorrhage time; *v* represents vasoconstriction time, and *c* represents coagulation time. From the *IP* values assayed, the lesions were divided into non-irritant (0–0.9), slightly irritant (1–4.9), moderately irritant (5–8.9), and severe irritant (9–21).

### 4.13. Statistical Analysis

All the findings were presented as mean ± standard deviation (SD). The data were assessed statistically with GraphPad Prism (version 8.0.1). One-way ANOVA followed by Tukey’s test was performed while considering the *p* values less than 0.05 as significant.

## Figures and Tables

**Table 1:** Photostability study of LEF and LEF-SLN dispersions.

Time (h)	LEF in Aqueous Tween^®^ 80		LEF-SLN Dispersions in Transparent Glass			LEF-SLN Dispersions in Amber Color Glass		
	pH ± SD	Total Drug Content (%) ± SD	pH ± SD	Total Drug Content (%) ± SD	Entrapment Efficiency (%) ± SD	pH ± SD	Total Drug Content (%) ± SD	Entrapment Efficiency (%) ± SD
0	6.63 ± 0.05	99.86 ± 1.62	5.08 ± 0.06	97.29 ± 1.43	65.57 ± 0.05	5.08 ± 0.06	97.29 ± 1.43	65.15 ± 0.04
3	7.21 ± 0.07	83.02 ± 1.55	5.51 ± 0.03	88.58 ± 1.88	65.25 ± 0.06	6.11 ± 0.03	92.40 ± 1.54	64.98 ± 0.14
6	6.57 ± 0.06	67.44 ± 1.79	5.47 ± 0.02	83.16 ± 1.39	64.94 ± 0.04	5.63 ± 0.02	86.98 ± 1.28	64.96 ± 0.06
9	6.48 ± 0.08	46.57 ± 1.64	5.39 ± 0.03	77.07 ± 1.54	64.44 ± 0.07	5.49 ± 0.04	85.41 ± 1.67	64.81 ± 0.07
10	6.40 ± 0.07	27.56 ± 1.82	5.35 ± 0.03	73.62 ± 1.76	64.16 ± 0.10	5.40 ± 0.03	84.15 ± 1.37	64.45 ± 0.11

**Table 2:** Visual appearance and pH of samples.

Samples	Visual Appearance	pH ± SD
Blank SLN dispersions	Translucent	3.26 ± 0.01
LEF-SLN dispersions	Translucent	3.51 ± 0.04
Blank SLN hydrogel	Smooth translucent	6.64 ± 0.02
LEF-SLN hydrogel	Smooth translucent	6.66 ± 0.04
LEF hydrogel	Clear	6.30 ± 0.01

**Table 3:** Percentage water loss and occlusion factor for LEF-SLN hydrogel, blank SLN hydrogel, LEF hydrogel, and control.

Time (h)	% Average Water Loss				Occlusion Factor, F		
	LEF-SLN Hydrogel ± SD	Blank SLN Hydrogel ± SD	LEF Hydrogel ± SD	Control ± SD	LEF-SLN Hydrogel ± SD	Blank SLN Hydrogel ± SD	LEF Hydrogel ± SD
24	9.85 ± 0.15	10.77 ± 0.44	38.15 ± 0.19	42.49 ± 0.11	76.82 ± 0.35 ^a^	74.66 ± 1.03 ^a^	10.20 ± 0.45
48	10.13 ± 0.81	10.95 ± 1.26	54.67 ± 0.32	68.16 ± 0.26	85.13 ± 1.19 ^b^	83.93 ± 1.85 ^b^	19.79 ± 0.46

Statistical data analysis from the one-way ANOVA followed by Tukey’s test for multiple comparisons, Occlusion factor, F: ^a^ *p* < 0.0001 (vs. 24 h LEF hydrogel), ^b^ *p* < 0.0001 (vs. 48 h LEF hydrogel). Values expressed as mean ± SD, n = 3.

## Data Availability

The data presented in this study are contained within the article.
